# Subdiaphragmatic Vagotomy With Pyloroplasty Ameliorates the Obesity Caused by Genetic Deletion of the Melanocortin 4 Receptor in the Mouse

**DOI:** 10.3389/fnins.2018.00104

**Published:** 2018-03-01

**Authors:** Ghazaul Dezfuli, Richard A. Gillis, Jaclyn E. Tatge, Kimbell R. Duncan, Kenneth L. Dretchen, Patrick G. Jackson, Joseph G. Verbalis, Niaz Sahibzada

**Affiliations:** ^1^Department of Pharmacology and Physiology, Georgetown University Medical Center, Washington, DC, United States; ^2^Department of Surgery, Georgetown University Medical Center, Washington, DC, United States; ^3^Department of Medicine, Georgetown University Medical Center, Washington, DC, United States

**Keywords:** vagotomy, weight loss, surgery, melanocortin, food intake, body weight, energy expenditure

## Abstract

**Background/Objectives:** We tested the hypothesis that abolishing vagal nerve activity will reverse the obesity phenotype of melanocortin 4 receptor knockout mice (*Mc4r*^−/−^).

**Subjects/Methods:** In two separate studies, we examined the efficacy of bilateral subdiaphragmatic vagotomy (SDV) with pyloroplasty in the prevention and treatment of obesity in *Mc4r*^−/−^ mice.

**Results:** In the first study, SDV prevented >20% increase in body weight (BW) associated with this genotype. This was correlated with a transient reduction in overall food intake (FI) in the preventative arm of the study. Initially, SDV mice had reduced weekly FI; however, FI normalized to that of controls and baseline FI within the 8-week study period. In the second study, the severe obesity that is characteristic of the adult *Mc4r*^−/−^ genotype was significantly improved by SDV with a magnitude of 30% loss in excess BW over a 4-week period. Consistent with the first preventative study, within the treatment arm, SDV mice also demonstrated a transient reduction in FI relative to control and baseline levels that normalized over subsequent weeks. In addition to the accompanying loss in weight, mice subjected to SDV showed a decrease in respiratory exchange ratio (RER), and an increase in locomotor activity (LA). Analysis of the white fat-pad deposits of these mice showed that they were significantly less than the control groups.

**Conclusions:** Altogether, our data demonstrates that SDV both prevents gain in BW and causes weight loss in severely obese *Mc4r*^−/−^ mice. Moreover, it suggests that an important aspect of weight reduction for this type of monogenic obesity involves loss of signaling in vagal motor neurons.

## Introduction

The vagus nerve is the major parasympathetic link between the gastrointestinal (GI) tract and the brain. Bilateral sub-diaphragmatic vagotomy (SDV), a surgical technique removing all vagal fibers including efferent (secretomotor) and afferent (sensory) fibers (Loeweneck and Holle, 1974; Nyhus et al., 1980; Powley et al., 1983; Laiwand et al., 1987) has uncovered a role for the vagus nerve in regulating body weight. In animal models, SDV abolishes obesity associated with bilateral electrolytic and knife-cut destruction of the ventromedial hypothalamus (VMH; Powley and Opsahl, 1974; Inoue and Bray, 1977; Rowland and Engle, 1978; Sawchenko and Gold, 1981; Sawchenko et al., 1981) a site shown to be to be critical to the hypothalamic obesity syndrome (Heinbecker et al., 1944; Gold et al., 1977; Aravich and Sclafani, 1983; Sims and Lorden, 1986; King, 2006).

In the case of VMH lesions, a unique enhancement of parasympathetic vagal nerve activity is produced that is associated with increased insulin, gastric acid secretion, gastric emptying, and gastrointestinal cell proliferation (Hales and Kennedy, 1964; Ridley and Brooks, 1965; Frohman and Bernardis, 1968; Frohman et al., 1969; Han and Frohman, 1970; Bernardis and Frohman, 1971; Inoue et al., 1977, 1983; Jeanrenaud, 1978; Berthoud and Jeanrenaud, 1979; Bray and York, 1979; Inoue and Bray, 1979, 1980; Sawchenko, 1979; Weingarten and Powley, 1980; Bray et al., 1981; Cox and Powley, 1981a; Bray, 1984; Yoshimatsu et al., 1984; Duggan and Booth, 1986; Kiba et al., 1992, 1993, 1996; Kintaka et al., 2009; Suzuki et al., 2014), which in the case of hyperinsulinemia and gastric hyperacidity is counteracted by SDV (Powley and Opsahl, 1974; Inoue and Bray, 1977; Berthoud and Jeanrenaud, 1979).

Similar changes in body weight, food intake, and autonomic nervous system function that accompany VMH lesions are also seen with impairments in the melanocortinergic system, a critical component of the brain's control of energy balance (Cone, 2005). The central melanocortin system's suppressive effects on food intake and body weight is mainly by its action via the melanocortin 4 receptor (*Mc4r*) in the PVN (Cone, 2005). Deletion of the *Mc4r* in mice fed a standard lab chow diet exhibit a mild obesity phenotype (Butler et al., 2001), which manifests as an aberrant state of energy balance characterized by disruptions in ingestive behavior (hyperphagia), a dysregulated metabolic phenotype (hyperglycemia, hyperinsulinemia, dyslipidemia), as well as massive hepatic steatosis when fed a high fat diet (Huszar et al., 1997; Marsh et al., 1999a; Rahmouni et al., 2003; Barb et al., 2004; Balthasar et al., 2005; Cone, 2005; Butler, 2006; Itoh et al., 2011).

Additionally, *Mc4r*^−/−^ mice that have not yet attained full obesity, exhibit reduced locomotor activity (Chen et al., 2000; Ste Marie et al., 2000; Butler et al., 2001) that is accompanied by a higher respiratory exchange ratio (Dean et al., 2009; Speakman, 2013). A higher RER value (0.9–1.0) indicates the reduced use of fatty acid oxidation for daily energy expenditure (EE), which is linked to an increased risk for weight gain and insulin resistance (Ravussin and Smith, 2002).

*Mc4rs* also impact food intake, gastric motility, and aspects of EE through signaling at the level of the brainstem nucleus of the solitary tract (NTS; Williams et al., 2002; Zheng et al., 2005; Skibicka and Grill, 2008, 2009; De Jonghe et al., 2011). A principal target of the NTS is the adjacent dorsal motor nucleus of the vagus nerve (DMV), where parasympathetic cell bodies of the efferent vagus nerve are distributed in a loosely viscerotropic pattern (Altschuler et al., 1989). Previously, we reported that stimulation of *Mc4rs* in the NTS inhibits output neurons of the DMV (Richardson et al., 2013). If activity in the melanocortin circuits leads to silencing DMV neurons (and hence a suppression of parasympathetic vagal outflow), then hyperactivity in these vagal efferent fibers, and the resultant autonomic profile (hyperinsulinemia and gastric hyperacidity etc.) would be expected to contribute to hyperphagia and obesity; similar to that seen in the VMH obesity syndrome. This rationale, coupled with the observation that *Mc4r* expression is seen in vagal fibers (likely originating from the DMV) that innervate the liver, stomach, and duodenum (Gautron et al., 2010), indicates that the observed changes in GI vagal efferent signaling may underlie the pathophysiological autonomic changes pivotal for the development and maintenance of obesity produced by the loss of the *Mc4r*.

Altogether, these observations suggest that the obesity produced by hypothalamic nuclei lesions (VMH and PVN) and the dysregulation of brainstem melanocortin pathway(s) in the homeostatic control of food intake, body weight, and EE share similar metabolic and/ or neuro-anatomical substrates that may engage the vagus nerve, To test this hypothesis, we performed SDV in obese *Mc4r*^−/−^ mice, and monitored their food intake and body weight. Our results show that SDV significantly prevents weight gain in *Mc4r*^−/−^ mice, as well as it effectively reduces body weight in this obese genotype. Furthermore, the results of the present study suggest that the efferent vagus nerve originating from the DMV serves as an important conduit for the central melanocortinergic system to regulate body weight.

## Materials and methods

### Animals

The animals used in this study were obese 3- to 5-months male homozygous loxTB *Mc4r*^−/−^ mice (stock number: 006414, Jackson Laboratories, Bar Harbor, Maine, United States; *n* = 22) and age-matched C57BL/6J (*n* = 6) mice for the first “preventative” study, and severely obese 8-month-old male homozygous loxTB *Mc4r*^−/−^ mice (*n* = 15) and C57BL/6J (*n* = 10) for the second “treatment study.” Homozygous mice exhibit severe obesity due to a loxP-flanked transcriptional blocking (loxTB) sequence that prevents normal endogenous gene transcription and translation from the endogenous locus. As such, homozygous mice are devoid of functional mRNA in all tested regions of the brain that endogenously express *Mc4r*. Animals used in this study were reared in an in-house breeder colony at Georgetown University and were bred from a homozygous breeder pair purchased from Jackson Laboratories. The control C57BL/6J mice were also purchased from Jackson Laboratories, and together with *Mc4r*^−/−^ mice were housed in a 12-h light/dark cycle under controlled temperature and humidity. All animals had *ad libitum* access to food and water except the night prior to surgery and during the recovery phase where the animals were pellet-deprived and fed a liquid diet of berry-flavored Ensure Enlive. All procedures were performed in accordance with the National Institutes of Health guidelines for use of animals in research and with the approval of the Georgetown University Animal Care and Use Committee.

### Feeding and weight measurements

Mice were acclimated to individual housing for at least 3 days prior to baseline measurement of their food intake (FI) and body weight (BW) over a 15 day period. All mice were provided access *ad libitum* to regular mouse chow that was pre-weighed (Low fat Purina Diet 5001; composition: 23.0% protein, 4.5% fat, 5.3% crude fiber, 49% carbohydrate, total digestible nutrient 76%, 3.04 kcal/g metabolizable energy). To assess FI, daily measurements were made between 3 and 5 p.m. by weighing each pellet manually and taking into account food spillage. FI was recorded for 4 weeks post-surgery.

### Surgical procedures: bilateral sub-diaphragmatic vagotomy

Prior to surgery, all animals were solid food-deprived overnight (18–24 h), while access to water and a berry flavored nutrition liquid (Ensure Enlive) was provided *ad libitum*. This was done to ensure as little solid food in the stomach as possible. All animals were anesthetized with isoflurane [induction 4%; maintenance 1.5–2% of minimum alveolar concentration (MAC)] and their body temperature was maintained at 37°C with an infrared heating lamp. Following adequate depth of anesthesia (as monitored via the toe pinch reflex), a midline abdominal incision (~1 cm) was made along the *linea alba* running ~1 cm caudal from the xiphisternum. The liver was retracted with a saline dampened cotton swab, and gentle traction was applied to the esophagus by lifting the stomach out of the peritoneal cavity using an umbilical tape wrapped around the gastric antrum. A curved 22-gauge glass rod was placed carefully under the esophagus to gently lift it and hold it in an exposed position. All identifiable vagal afferent and efferent fibers running along the esophagus orad to the esophagogastric junction (~1 cm) were visualized with the aid of a surgical microscope (Bausch & Lomb, Inc.) and excised with a microsurgical hook having an interior cutting edge (Circon, Corp.). In each case, a vagal segment (as long as possible) was exposed and isolated, which was then excised. This consisted of the removal of all four major components of the abdominal vagus (celiac, hepatic, gastric, and both ventral and dorsal vagal trunks). In addition, all neural tissue surrounding the esophagus immediately beneath the diaphragm was excised with a microsurgical hook to capture any residual vagal branches. In all animals that received a vagotomy, extreme care was taken to avoid any damage to the subdiaphragmatic esophagus. As a result, both food intake and defecation were similar in these animals to those of sham-vagotomized and pyloroplasty alone controls [Note: Signs of dysphagia-associated damage to the upper esophageal sphincter or pharynx (abnormal licking, mastication patterns, or abnormal breathing) were not observed in any animals that underwent vagotomy or sham-vagotomy]. After this procedure, the umbilical tape and glass rod were removed. For sham-operated animals, the vagus nerves were similarly exposed but not cut, and the stomach received gentle traction with the umbilical tape.

In our initial studies of mice that underwent bilateral SDV, none of the mice survived more than a few days due to marked gastric distention and pyloric stenosis. To circumvent this problem, we performed a modified Heineke-Mikulicz pyloroplasty to facilitate gastric drainage and relieve pyloric stenosis.

### Heineke-mikulicz pyloroplasty

After bilateral subdiaphragmatic vagotomy, a pyloroplasty procedure was subsequently performed in the anesthetized mice. To accomplish this, the gastric antro-pyloro-duodenal region was gently exposed and lifted with a 22-gauge bent gavage needle and a fine forceps were placed under the pylorus to stretch it. A longitudinal incision (~2–4 mm) was made through the pylorus, extending from the distal antrum to the proximal duodenum. Four (5-0 Vicryl) sutures were placed in the pyloric tissue such that by closing the incision transversely, the outlet diameter of the pylorus was increased. A small piece of absorbable gelatin sponge was placed over the pyloroplasty and secured in place with the remaining thread of the last pyloric tissue suture to further ensure against possible leakage of intestinal contents into the peritoneal cavity. The duodenum/stomach was then placed back in the peritoneal cavity and the abdominal incision closed in a two-step procedure using a 5-0-vicryl suture. Postsurgical medication consisted of Baytril (5 mg/kg SC) for infection prevention, 1cc SC saline for prophylactic hydration, and Rimadyl (5 mg/kg SC) for analgesia. No gastric distention was observed in animals undergoing this procedure, indicating that the antro-pyloro-duodenal junction remained patent. For pyloroplasty only animals, the subdiaphragmatic vagal trunks were similarly isolated and exposed but not excised; only a pyloroplasty was performed.

### Post-recovery phase

For 1 week, all animals were monitored for piloerection, ptosis, porphyrin, hunching, mobility, and ruffled coat. Any animal displaying any of these adverse effects was removed from the study. To minimize post-surgery disturbances of intestinal motility, during the post-recovery phase, animals were maintained on Ensure Enlive berry-flavored nutrition drink.

### Histological verification of completeness of bilateral sub-diaphragmatic vagotomy

After the completion of both studies, each animal received a single intraperitoneal injection of FluoroGold (0.8 mg/0.4 ml saline; Fluorochrome, Denver, CO). One week after injection, all animals were euthanized with an intraperitoneal administration of pentobarbital (200 mg/kg) and perfused via the ascending aorta with a phosphate buffered saline wash (0.1 M, pH 7.4) followed by a buffered 4% paraformaldehyde (0.1 M, pH 7.4) fixative delivered over a 15 min period. The brains were removed and placed in fixative (4% buffered paraformaldehyde-20% sucrose) for at least 24 h. After cryoprotection, the brainstem was separated and cut on a cryostat (Leica, 1500) into 50 μm coronal sections, which were mounted on slides for later imaging using episcopic fluorescence microscopy. Each brainstem section (~9 sections/slide; total slides = 5/brainstem) was examined for FluoroGold label in the DMV. The presence of fluorescent label in DMV neurons was accepted as a marker of incomplete vagotomy. Only animals with complete or no vagotomy were included in the study.

### Fat pad dissection

After the completion of both studies, animals were euthanized (see above) and the fat pads dissected from the following compartments: mesenteric, peri-renal, sub-scapular, and epididymal. The wet weights of the different fat pads were then weighed.

### Metabolic chamber recordings of indirect calorimetry

Adult male *Mc4r*^−/−^ mice (*n* = 13) were acclimated in metabolic chambers (PhenoMaster: TSE Systems, Bad Homburg, Germany) for 3 days before the start of recordings. After acclimation for 3 days, oxygen (VO2) consumption and carbon dioxide (VCO2) production were acquired every 10 min for 72 h via the PhenoMaster software. Metabolic parameters that include energy expenditure (EE) and respiratory exchange ratio (RER = VCO2/VO2) were calculated by algorithms native to the supplied acquisition software. A detailed description and protocol of these can be obtained directly from the manufacturer or found on the website (www.tse-systems.com). Briefly, the software generates 3 types of calorimetric datasets of which the first takes into account the animal's body weight and the second takes into account the lean body mass (LBM), calculated from a user-defined power of the animal's weight in a range of 0.001–0.999. In the third set, neither the animal's weight or LBM is taken into consideration.

In analyzing the data, we took into consideration which subset of the data generated by the PhenoMaster software would most accurately reflect the calorimetric parameters associated with our experimental conditions. It is well-documented that indirect calorimetry to compare EE among animals that differ in body weight has inherent inaccuracies (Arch et al., 2006; Butler and Kozak, 2010; Tschöp et al., 2012). First, differences in body weight are usually associated with differences in tissue distribution, making it difficult to calculate their specific contribution. For example, adipose tissue may have low EE in obese animals and although the metabolic rate of fat tissue may be low, it is not zero and may represent a relatively high proportion of the animal's total mass. Second, adipose tissue releases adipokines, notably leptin that affect EE in other tissues. Third, bigger animals with their larger body mass are widely accepted to expend more energy (Felig et al., 1983; Arch et al., 2006). Hence, to avoid these potentially confounding variables, many investigators choose to normalize VO_2_ using body weight or, preferably, to LBM, which many investigators feel accurately reflects total EE (Tschöp et al., 2012; Speakman, 2013). It is worth noting, however, that normalization to LBM has its own inherent flaws since the lean tissue is not distributed evenly in all compartments and, as a result, LBM does not compare isometrically with total body mass (Arch et al., 2006; Speakman, 2013). In the present study, since a body composition analyzer (EchoMRI) was not available to us, we did not measure LBM. Additionally, a ratio-based method for normalizing EE to LBM is not presented. We do present EE normalized to body weight [kcal/h/kg], as well as non-normalized EE data [kcal/h], because of substantial differences in the body size of the study animals.

### Data analysis

Data are presented as mean ± SEM. In all cases, *p* < 0.05 was the criterion used to determine statistical significance. Statistical analysis of the fat pads was performed using a one-way analysis of variance (ANOVA) and unpaired-*t-* tests, whereas body weight and cumulative food intake were analyzed employing a two-way repeated measure ANOVA and Bonferroni's multiple comparison *post-hoc* tests.

To analyze the metabolic data, a linear regression model was employed to determine the relationship of EE (kcal/hour) to body mass within and across groups. EE was averaged with respect to time and the resulting averaged EE values were fitted using a linear regression model with body weight as an independent variable. Locomotor activity (LA) was measured across the long axis of the cage by an array of infrared sensors. Activity data is reported here as the combination of ambulatory activity (mouse crosses two adjacent beams) and fine activity (i.e., grooming where the mouse crosses the same beam twice). Normalized EE (kcal/h/kg), RER, and LA were analyzed for both dark and light cycle phases using repeated measures one-way analysis of variance (ANOVA) with *post-hoc* analysis of the data using the Tukey multiple comparison test. [**Note**: for the dark cycle LA measurements, light cycle phase data was filtered to only include data points where the animals showed no LA (i.e., animals are presumed to be at rest)]. The 24 h EE, RER, and LA data was computed from the first 24 h of indirect calorimetry data.

## Results

### Histological evidence of completeness of subdiaphragmatic vagotomy

**Figure 1:**
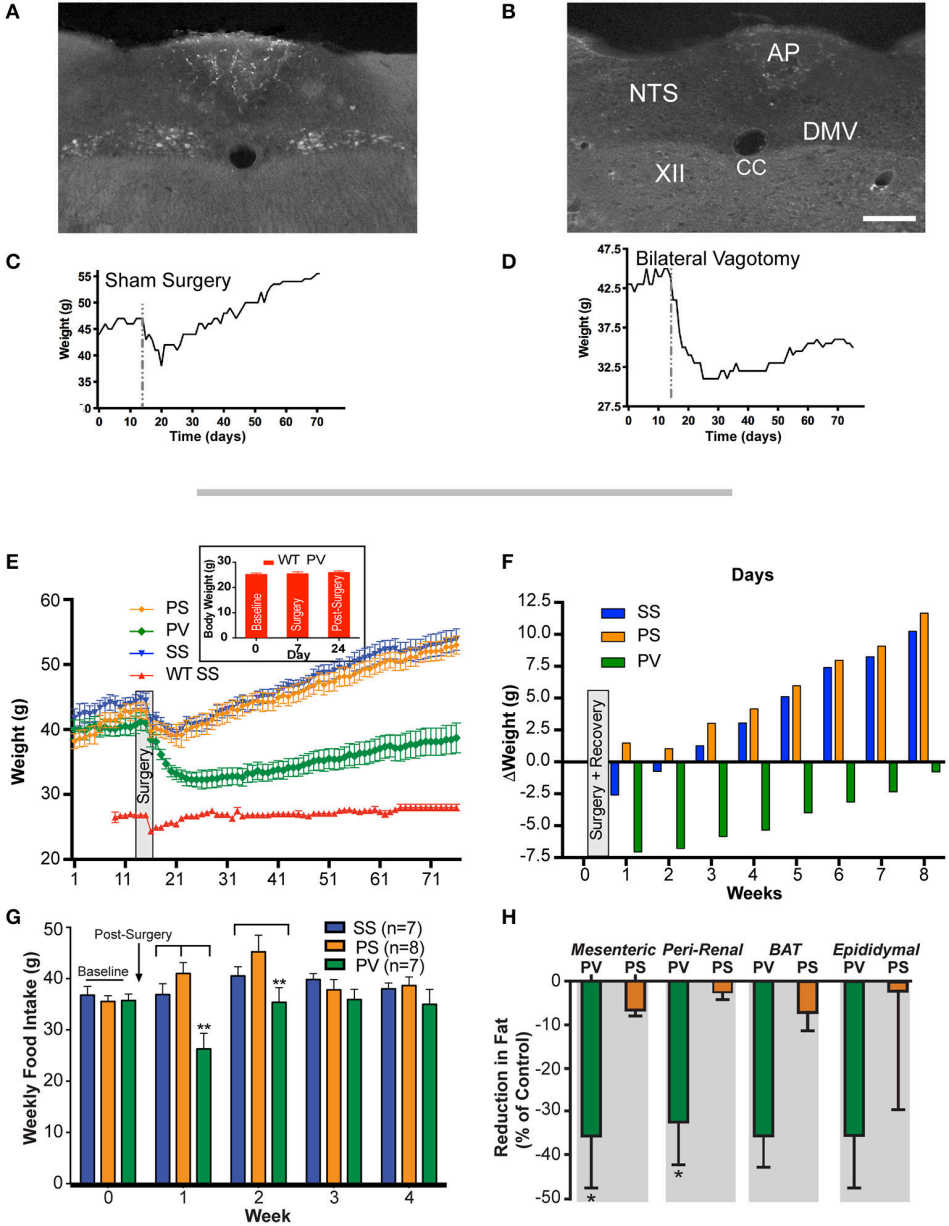
Weight gain is prevented by subdiaphragmatic vagotomy (SDV) in not yet fully obese *Mc4r*^−/−^ mice (< 45 g). **(A)** Representative photomicrograph showing the presence of FluoroGold label in DMV neurons of a not yet fully obese *Mc4r*^−/−^mouse with intact bilateral subdiaphragmatic vagus nerves (sham SDV and sham pyloroplasty; SS). **(B)** Representative photomicrographs showing the absence of FluoroGold label in DMV neurons of not yet fully obese *Mc4r*^−/−^mouse with complete bilateral SDV (SDV and pyloroplasty; PV). **(C)** Representative associated weight curve following sham surgery. **(D)** Representative associated weight curve following vagotomy and pyloroplasty surgery. **(E)** Graph showing the effect of bilateral SDV and pyloroplasty on body weight. Male mice (*Mc4r*^−/−^ or wild type; *n* = 6–8 *per* treatment group) were weighed daily after undergoing: *sham* SDV and *sham* pyloroplasty (SS, blue), *sham* SDV and pyloroplasty (PS, orange), SDV and pyloroplasty (PV, green), wild type *sham* SDV and *sham* pyloroplasty (WT SS, red) or wild type SDV and pyloroplasty (WT PV, insert graph). Data are expressed as the mean ± SEM of the body weight (g). **(F)** Graph illustrating the effect of subdiaphragmatic vagotomy and pyloroplasty on the average change in weekly body weight of *Mc4r*^−/−^ mice, relative to baseline (*n* = 6–8 per treatment group). **(G)** Graph showing cumulative weekly food intake in all groups fed on a normal diet. Statistical significance in weekly food intake was obtained by comparing treatment group PV with control groups (PS and SS). **(H)** Bar graphs showing the percent reduction in adipose tissue in PV and PS *Mc4r*^−/−^ mice (*n* = 6–8 per treatment group) fed a normal diet (values are expressed as mean % change (reduction) in relation to the wet weight of adipose tissue in the SS controls). ^*^p < 0.05 and ^**^p < 0.01 criterion for statistical significance using a one-way or two-way ANOVA and with *post-hoc* comparison tests (Bonferoni and Tukey). XII, hypoglossal nucleus; AP, area postrema; CC, central canal; DMV, dorsal motor nucleus of the vagus; NTS, nucleus tractus solitarius. Scale bar = 100 μm **(A,B)**.

The effectiveness and completeness of the SDV surgery in these studies was verified by the retrograde tracer Fluro-Gold (IP). As expected, in sham operated mice (sham SDV and sham pyloroplasty; SS group; *n* = 7), Fluoro-gold labeled neurons were abundantly present in the dorsal motor nucleus of the vagus (DMV; Figure 1A) compared to those undergoing SDV and pyloroplasty where labeled neurons were absent in the DMV (PV group; *n* = 7; Figure 1B). The presence and absence of fluorescence label in the DMV in the two groups attest to the intactness of the vagus nerve in the SS group and the completeness of SDV in the PV group, respectively. Furthermore, these observations were corroborated by differences in body weight (BW) between the two groups of mice. In the SS group, following an initial reduction in post-surgery, the weight exceeded that of pre-surgical values (Figure 1C) which in the case of the PV group did not recover to baseline levels (Figure 1D).

### Subdiaphragmatic vagotomy prevents weight gain in *Mc4r^−/−^* mouse

As indicated in the methods, bilateral SDV *per-se* is lethal in mice, therefore for it to be successful, it has to be performed in conjunction with a pyloroplasty. Hence, to assess the effect of SDV as a preventative measure for *Mc4r*^−/−^ deficient obesity, mice were assigned to three groups. These groups underwent the following treatments: **Group 1**, sham SDV and sham pyloroplasty (SS, *n* = 7); **Group 2**, pyloroplasty and sham SDV (PS, *n* = 8); and, **Group 3**, pyloroplasty and SDV (PV, *n* = 7). Prior to surgery, (see section Materials and Methods), initial baseline weights (obtained over a 2 week period) of the three groups of mice were 44.0 ± 1.1, 41 ± 1.2, and 40.0 ± 1.0 g, respectively (Figure 1E). After surgery, all mice were placed on a liquid diet for 7 days. Their normal diet was re-established on post-surgery Day 8. From thereon, body weight (BW) was monitored daily and plotted over an 8-week period (Figure 1E). As can be noted, the SS and PS groups after initially losing weight following surgery gained it at a fairly constant rate. At the end of the 8-week period, the SS and PS groups had attained weights of 54 ± 1.4 and 53 ± 1.3 g, respectively. Mice that underwent both pyloroplasty and SDV (PV) lost weight initially but then gained it, though at a much slower rate than those mice in the SS and PS groups. Indeed, at the end of the 8-week monitoring period, the PV group weighed only 38.7 ± 2.3 g, a value significantly less than the corresponding weights of the SS and PS groups (*p* < 0.05). Instead, the PV group of mice began to return to the weight that they had prior to surgery (Figure 1E). This represents a 2.5% decrease in BW over the 8-week period compared to an overall increase in BW that was seen for both the SS and the PS groups (22.7 and 29.2% respectively). More importantly, the >20% increase in BW over an 8-week period that would have been expected in *Mc4r*^−/−^ mice (as seen in SS and PS controls) was blunted in the PV group.

The change in weight of the three groups over the duration of the post-surgical monitoring period is depicted in Figure 1F. As can be noted, the SS and PS groups gained 10.2 g and 11.6 g respectively, while the PV group lost 0.8 g. Inspection of the data clearly indicates that pyloroplasty alone was not responsible for the ability of SDV to cause the *Mc4r*^−/−^ obese mice to gain weight at a much slower rate than the SS and PS groups. Instead, the slower rate of weight gain in the PV group appeared to be due entirely to the SDV procedure.

Examination of the data also raised the question as to whether the efficacy of SDV, in addition to preventing obesity as seen in Figure 1E, is robust enough to restore the weight of *Mc4r*^−/−^ mice to that of normal age-matched C57BL/6J mice (wild-type; WT). In other words, is SDV able to reverse the obesity phenotype associated with the *Mc4r* deficiency and bring the weight of the mice back to that of the normal weight of the control background strain (C57BL/6J). To address this question, we studied a fourth group of mice, namely a WT group (*n* = 6) that underwent both sham pyloroplasty and sham SDV. Their body weights at baseline, during recovery after surgery, and over the 8-week monitoring period are shown in Figure 1E (WT SS group). The starting average BW of this group was 27.0 ± 0.4 g. As can be noted from Figure 1E, except for an initial drop in weight during sham surgery, this group of mice steadily maintained their body weights over the 8-week period of observation (28.3 ± 0.6 g). This value was not significantly different from their baseline value, but clearly less than the corresponding value of the PV group (*p* < 0.05) at any time point. These data indicate that SDV while having a robust effect to counter the weight gain observed in the *Mc4r*^−/−^ mice, did not restore the BW of these animals to normal. With regards to the general well-being of the PV mice, each mouse appeared in good health and, based on general behavior, could not be distinguished from mice in the other groups; except by body size. Moreover, the fecal output of this group was of normal volume and consistency and could not be distinguished from those of other groups; neither could their eating, drinking, or urinating.

Solid food intake (FI) data recorded over a 4-week period in the three *Mc4r*^−/−^ groups are presented in Figure 1G. As is evident, cumulative weekly FI of the PV group was significantly less (*p* < 0.05) than that of the SS and PS groups in the first week after post-surgical recovery and significantly less (*p* < 0.05) than that of the PS group in the second week after recovery. However, due to the fact that FI of the PV group normalized to that of both controls in subsequent weeks, no overall decrease in FI was observed.

Further examination of the data raised the critical question as to whether or not the weight loss associated with SDV in the *Mc4r*^−/−^ mouse may be confounded to non-specific effects of the surgery itself that could occur in any mouse model independent of genotype. To address this question, we studied a separate group of WT mice (*n* = 10) that underwent both vagotomy and pyloroplasty (PV). Their body weights at baseline, during recovery after surgery, and 17 days post-surgery are shown in Figure 1E (insert graph, WT PV group). The starting average BW of the group at baseline was 25.1 ± 0.68 g, whereas, on post-surgery day 17, the average weight of this group of mice was 25.9 ± 0.67 g. As can be noted from Figure 1E (insert graph) there was no statistically significant difference in body weight of these mice at baseline compared to 17 days post-surgery (*p* > 0.05; Figure 1E insert graph). These data indicate that SDV while having a robust effect on BW in *Mc4r*^−/−^ mice, is not effective in reducing BW in age-matched normal weight WT mice. In fact, in both the prevention and reversal studies using the obese *Mc4r*^−/−^ mouse, robust weight loss was seen 17 days post-surgery in the PV group. In the “prevention” study, these mice lost 20.2% of their baseline weight (see Figure 1E), while in the “reversal” study, they lost 30.3% of their initial excess weight (**Figure 3A**). The data demonstrate that weight loss associated with SDV in the *Mc4r*^−/−^ mouse is not due to non-specific effects of the surgery itself, but rather due to the underlying influence of the *Mc4r*^−/−^genotype.

### Effect of subdiaphragmatic vagotomy on fat pad weight and on snout-to-anus length in *Mc4r^−/−^* mice

**Figure 2:**
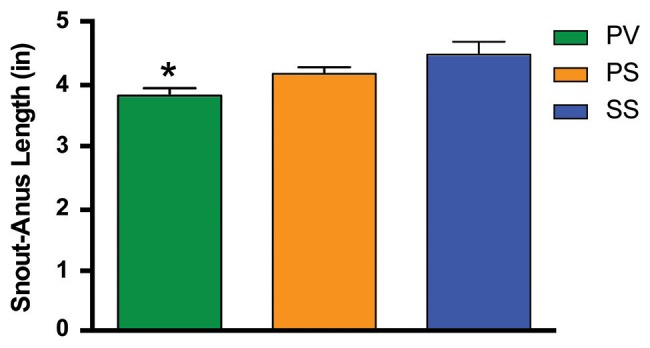
Bar graph showing the effect of bilateral subdiaphragmatic vagotomy + pyloroplasty on snout-to-anus length in not yet fully obese *Mc4r*^−/−^ (*n* = 6–8 per treatment group) fed a normal diet. ^*^p < 0.05 criterion for statistical significance using a one-way ANOVA and Tukey *post-hoc* comparison test.

We also determined whether SDV would prevent white adipose tissue (WAT) accumulation in *Mc4r*^−/−^mice. Fat pads were dissected from the visceral compartments in the 3 groups of mice. In the PV group, the weight of WAT as a percentage of that in the SS controls (mesenteric = 2.18 ± 0.13 g; peri-renal = 1.34 ± 0.09 g; epididymal = 2.7 ± 0.21 g; brown adipose tissue = 0.72 ± 0.10 g) was significantly less in both the mesenteric (MES) and peri-renal (PR) sub-compartments (*p* < 0.05; Figure 1H). In contrast, the weights of the fat pads taken from the PS was not different from that of the SS group. Sub-scapular brown fat (BAT) and epididymal fat pad (EPI) in the PV group was not statistically significant from that of the other two groups (Figure 1H). In addition, we measured the snout-to-anus length (an indicator of body growth) as it known to increase in *Mc4r*^−/−^obese mice (Huszar et al., 1997); SDV was found to significantly blunt this end-point, whereas pyloroplasty, *per-se*, had no effect on linear growth (*p* < 0.05; Figure 2).

### Subdiaphragmatic vagotomy treatment reverses obesity in *Mc4r^−/−^* mice

**Figure 3:**
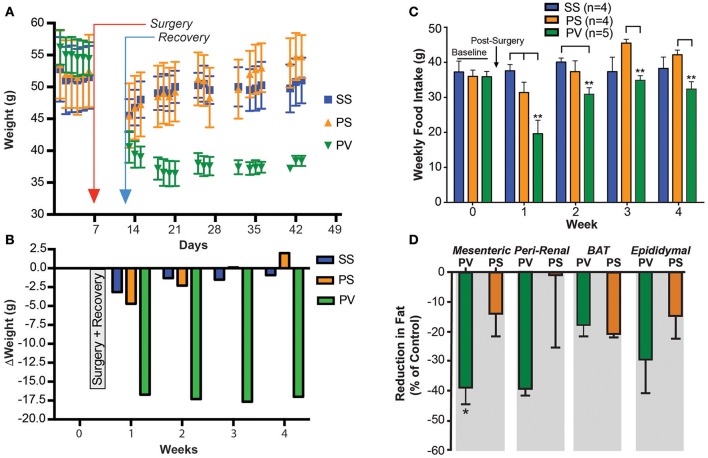
Subdiaphragmatic vagotomy treatment reduces body weight in severely obese *Mc4r*^−/−^ mice (> 50 g). **(A)** Graph showing the effect of bilateral subdiaphragmatic vagotomy and pyloroplasty on body weight. Male mice (*Mc4r*^−/−^, *n* = 4–5 per treatment group) were weighed periodically after undergoing: *sham* subdiaphragmatic vagotomy and *sham* pyloroplasty (SS, blue), *sham* subdiaphragmatic vagotomy and pyloroplasty (PS, orange), or subdiaphragmatic vagotomy and pyloroplasty (PV, green). Data are expressed as the mean ± SEM of the body weight (g). **(B)** Shows the effect of subdiaphragmatic vagotomy and pyloroplasty on the mean weekly change in body weight, relative to baseline (*n* = 4–5 per treatment group. **(C)** Graph showing cumulative weekly food intake in all groups fed on a normal diet. Statistical significance in weekly food intake was obtained by comparing treatment group PV with control groups (PS and SS). The PS group in weeks 3 and 4 exhibits increased FI relative to baseline levels whereas the PV group did not. **(D)** Bar graphs showing the percent reduction in adipose tissue in PV and PS *Mc4r*^−/−^ mice (*n* = 4–5 per treatment group) fed a normal diet [Values are expressed as mean % change (reduction) in relation to the wet weight of adipose tissue in the SS controls]. ^*^p < 0.05 and ^**^p < 0.01 criterion for statistical significance using a two-way ANOVA and Tukey *post-hoc* comparison test (an unpaired *t*-test was used for fat pad analysis).

Our purpose was to determine whether SDV could reverse the established obesity in the *Mc4r*^−/−^mouse. Mice had already become obese before SDV treatment was performed. Prior to surgery, initial baseline weights (obtained over a 1 week period) were 51 ± 0.3 g (SS), 52 ± 0.3 g (PS), and 55 ± 0.3 g (PV), respectively (Figure 3A). After surgery, all mice were placed on a liquid diet for 7 days. Their normal diet was re-established on post-surgical day 8. From thereon, body weights were monitored daily and plotted over a 4-week period. As can be noted, the SS and PS groups initially lost weight after surgery, but gained it back over time (Figure 3A). At the end of the 4-week period, the SS and PS groups had attained weights of 51 ± 3.6, 55 ± 3.4 g, respectively. Obese mice that underwent both pyloroplasty and SDV (PV group) lost weight initially, and then continued to lose weight over the 4-week period of the study (Figure 3A). Indeed, at the end of the 4-week monitoring period, the PV group weighed only 38.4 ± 0.8 g, a value significantly less than the corresponding weights of the SS and PS groups (51.0 ± 3.58 and 54.8 ± 3.4 g, respectively; *p* < 0.05). The change in weight of the 3 groups over the duration of the post-surgical monitoring period is depicted in Figure 3B. As can be noted, at the end of the 4-week monitoring period, the SS and PS groups had returned to their baseline weights while the PV group did not; they had lost 17 g from their initial baseline weight.

Solid food intake (FI) recorded weekly for the three *Mc4r*^−/−^groups are presented in Figure 3C. Similar to the prevention study (see Figure 1G), FI in the PV group of mice was significantly less (*p* < 0.05) than that of the SS and PS groups at post-surgical week 1 (week 3 of the overall study). In addition, FI of the PV group was reduced relative to the SS group during week 2. This is not reflective of an overall decrease in FI since in the PV group it normalized to that of the SS groups in the subsequent weeks (week 3 and 4). Interestingly, although FI for the PV group was not significantly reduced relative to the SS group during weeks 3 and 4, it was significantly less than that of the PS group (*p* < 0.05; Figure 3C). Furthermore, it should be noted that that the PS group increased their FI relative to baseline in weeks 3 and 4 (Figure 3C).

We also determined whether SDV would reverse WAT accumulation in fully obese *Mc4r*^−/−^mice. Fat pads were taken from the visceral components in the 3 groups of mice and weighed. The WAT content in PV group as a percentage of that present in the SS control tissue (MES = 1.8 ± 0.4 g; PR = 1.59 ±0.5 g; EPI = 2.02 ± 0.2 g; BAT = 0.3 ± 0.1 g) was significantly less in the MES sub-compartment (*p* < 0.05; Figure 3D). In the PR and EPI sub-compartments, the WAT content in the PV mice was not significantly different from that of the SS group, as was that of BAT, which was similar to those of the PS and SS groups (Figure 3D). Additionally, no significant differences were found in fat pads weights between the two control groups (PS and the SS group). The snout-to-anus length, reflective of body growth, was also unaffected by SDV in these fully obese animals (data not shown).

Data obtained from studying SDV as prevention and as a treatment strategy against *Mc4r*^−/−^ mouse obesity demonstrates a robust anti-obesity effect. The mechanisms for the anti-obesity effect initially include a transient reduction in FI (Figures 1G, 3C). However, the reduction in FI did not persist, which suggested to us that the SDV effect on BW maybe due to an increase in energy expenditure (EE). To determine this, we undertook a series of studies to measure the influence of SDV on metabolic parameters that are reflective of EE.

### Effect of subdiaphragmatic vagotomy on parameters of EE in fully obese *Mc4r^−/−^* mice

**Figure 4:**
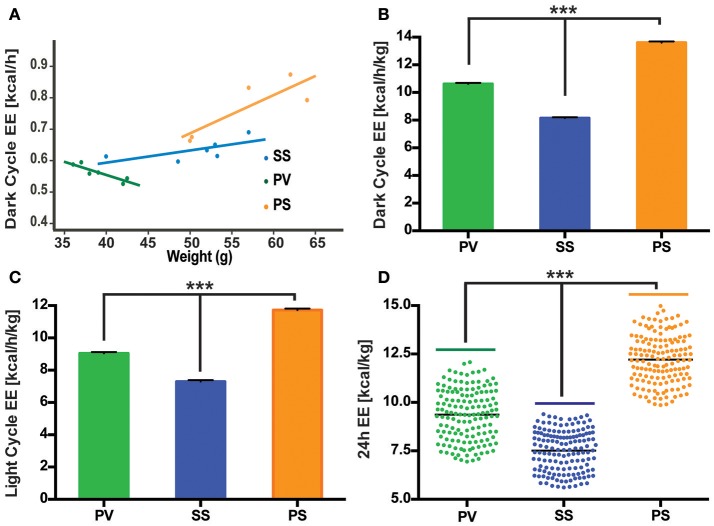
Bilateral subdiaphragmatic vagotomy influences EE in fully obese *Mc4r*^−/−^ mice. **(A)** Graph showing the relationship of EE (expressed in kcal/h) to body mass (*n* = 4–5 *per* treatment group). Data are presented as a linear regression plot. **(B,C)** Graphs showing mean EE (expressed in kcal/h/kg; *n* = 5–7 *per* treatment group) in dark and light cycles, respectively. **(D)** Vertical dot plot of 24 h EE data expressed as kcal/kg with mean (black bar). ^***^p < 0.001 criterion for statistical significance.

Metabolic parameters associated with EE were analyzed (see Materials and Methods) in relationship to BW in 3 groups of fully obese adult *Mc4r*^−/−^ mice as described above (SS, PS, and PV). We particularly focused on this relationship during the dark cycle, which is correlated with increased activity in rodents. While there was no significant correlation between BW and EE in the SS control group, animals undergoing SDV (PV group) showed an inverse linear relationship (*p* = 0.014, slope = −0.009, 95% confidence interval = [−0.014, −0.003], *R*^2^ = 0.81; Figure 4A). The decrease in BW in the PV group was associated with an increase in EE. This contrasts with the relationship of EE to BW in animals subjected to pyloroplasty and sham vagotomy (PS group). In the PS group (see Figure 4A), there was an indication of a positive correlation between EE and BW indicating that as they became heavier, the likelihood of their energy consumption also increased (*p* = 0.07, slope = 0.01, 95% confidence interval = [0.002, 0.026], *R*^2^ = 0.71). Despite the fact that there was no significant correlation in either control group, it is worth noting that both groups exhibited a positive relationship between BW and EE indicating that as they got heavier, they expended more energy. This was in contrast to the PV group that exhibited a negative relationship between the two variables. We also employed linear regression analysis to data obtained from the aforementioned three groups during the light cycle. No significant differences were observed in the relationship of EE to BW in all three groups (data not shown).

While body size and composition influences EE, there is no consensus on the best way to normalize VO_2_ or VCO_2_ data (Butler and Kozak, 2010; Kaiyala et al., 2010). We chose to present the data as normalization of EE to BW in the light and dark cycles to facilitate comparison to our the linear regression data (Figure 4A). As illustrated in Figures 4B–D, mean normalized EE [kcal/h/kg] was analyzed for the three groups in the dark and light cycles, as well as the first 24 h cycle of indirect calorimetry recording. As can be seen, PV mice displayed an increase in normalized EE relative to the SS control group in both the dark, light and 24 h cycle (Figures 4B–D). However, the mean normalized EE of the PS group (Figures 4B–D) was the highest of the three groups despite showing no weight loss (Figures 1E,F, 3A).

**Figure 5:**
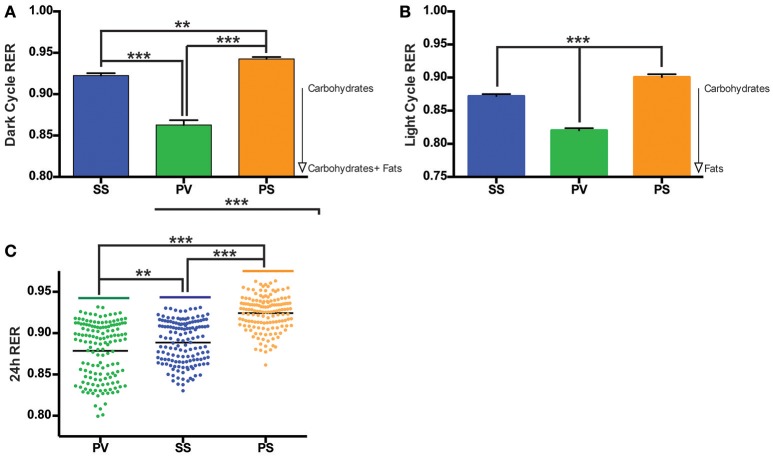
Bilateral subdiaphragmatic vagotomy lowers respiratory exchange ratio (RER) in fully obese *Mc4r*^−/−^ mice. **(A,B)** Bar graphs of the mean RER, *n* = 5–8 *per* treatment group during the dark cycle (averaged into 100 min bins to reduce clutter) and light cycle respectively. **(C)** Vertical dot plot of 24 h RER with mean (black bar). RER data was sampled at consecutive 10-min intervals over the course of the monitoring period. ^**^p < 0.01, ^***^p < 0.001 criterion for statistical significance.

Mean respiratory exchange ratio (RER) was collapsed over time and analyzed for the three groups. In comparison to the SS and PS groups, the overall RER of the PV group was lower (*p* < 0.05; Figures 5A,B) during both the dark cycle (12 h) and light cycle (4 h). The RER for the 24 h cycle is shown in Figure 5C.

**Figure 6:**
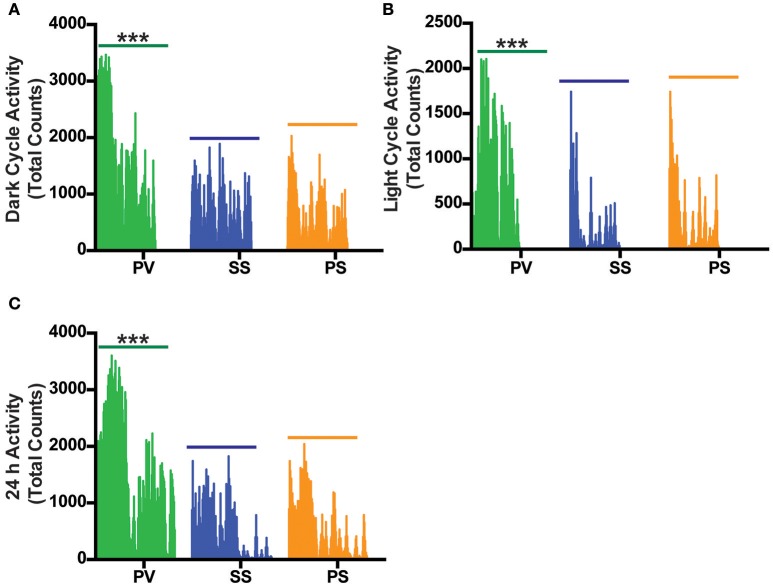
Bilateral subdiaphragmatic vagotomy increases total activity in fully obese *Mc4r*^−/−^ mice. **(A,B)** Vertical bar plot of total activity (combination of axial and fine movements) during the dark and light cycle acquired over an 18–28 h period that was sampled at 10-min intervals; *n* = 5–8 *per* treatment group. **(C)** Vertical bar plot of 24 h LA activity. ^***^p < 0.001 criterion for statistical significance.

Locomotor activity (LA; a combination of axial/ambulatory and fine movements) during the light cycle (collapsed over ~18 h) and dark cycle (collapsed over ~ 28 h) revealed a significant overall increase in LA in the PV group relative to the SS and PS groups with no change seen between either of the control group (*p* < 0.05; Figures 6A,B). A representative graph of the initial 24 h LA cycle is shown in Figure 6C.

## Discussion

Suppression of melanocortin signaling in the brain may represent an important mechanism responsible for hypothalamic lesion-induced obesity, which has been shown to engage the vagus nerve (Powley and Opsahl, 1974; Inoue and Bray, 1977; Cox and Powley, 1981a,b; Sawchenko and Gold, 1981; Sclafani et al., 1981; Sims and Lorden, 1986). In testing the notion that the obesity rising from lesions of both the VMH and PVN is due to the loss of a common molecular substrate (i.e., the *Mc4r*), we assessed the impact of SDV on *Mc4r* deficient obese mice in two separate experiments. We performed SDV as a “preventative” measure in 3- to 5-month-old *Mc4r*^−/−^ mice that had not yet attained full obesity (< 45 g) and as a “reversal” measure in 8-month-old *Mc4r*^−/−^ mice that were severely obese (>50 g). Findings from both studies reveal that SDV exerted a robust anti-obesity effect. On average, the *Mc4r*^−/−^ mice lost ~14 grams excess body mass (EBM) which represents 25% of the baseline weight of their obese phenotype.

Our results suggest that SDV is able to counteract the suppression of Mc4r signaling to create a state of negative energy balance at the level of the DMV. We suggest that activity in the melanocortin neural circuits eventually inhibits DMV neurons, which results in the suppression of vagal efferent output to GI tract (Gillis et al., 1989; Berthoud et al., 1991), liver (Sawchenko and Friedman, 1979), pancreas (Woods and Porte, 1974), and adipose tissue (Kreier and Buijs, 2007). Conversely, interruption of melanocortin neural circuits should increase efferent vagal activity, as is evident by the increase in hyperinsulinemia in *Mc4r*^−/−^ mice (Huszar et al., 1997; Rossi et al., 2011). Thereby, SDV or surgical ablation of the DMV would compensate for the lack of melanocortin signaling by effectively dampening the elevated parasympathetic outflow toward autonomic balance. Indeed, elimination of efferent vagal activity by bilateral SDV has been shown to reduce BW to normal in animals with VMH lesions (Powley and Opsahl, 1974; Inoue and Bray, 1977; King et al., 1978). Overall, the anti-obesity effect of SDV in *Mc4r*^−/−^ mice fits well with our view that the DMV serves as an important conduit of central melanocortin circuitry by which the brain conveys information to the periphery.

What led us to focus on the DMV as the main site of action whereby the physiological effects of suppressed melanocortin signaling (leading to an obese phenotype) could be “reversed” were the findings of others, who showed that: (1) melanocortin neural circuits convey information into the NTS (Zheng et al., 2005, 2010; Berthoud et al., 2006; Blevins et al., 2009), (2) pro-opiomelanocortin (POMC) gene transfer into the NTS or its activation by melanocortin agonists ameliorates diet-induced obesity in rodents (Li et al., 2007; Zhang et al., 2010), and (3) the NTS sends major efferent projections to the DMV (Rogers et al., 1980). These observations, together with our finding, (Richardson et al., 2013) that activation of *Mc4rs* in the NTS results in inhibition of the DMV and vagal outflow to the stomach, compelled us further to evaluate the effect of SVD on *Mc4r*^−/−^ associated obesity. Our present findings with SDV in the *Mc4r*^−/−^ mouse not only strengthens the link between brainstem melanocortin signaling in regulating efferent vagal nerve activity, but are also consistent with the view that release of α-MSH in the NTS via activation of a hypothalamic-POMC-NTS circuit causes amplification of satiety signals arriving from the stomach (Berthoud et al., 2006; Zheng et al., 2010), thereby suppressing hunger. Although how this is accomplished is speculative, our view is that α-MSH blocks the tonic inhibition of GABAergic projection neurons in the NTS that innervate the vagal output neurons of the DMV (Richardson et al., 2013). This “decoupling” of NTS projection neurons from ‘local’ inhibition (Herman et al., 2009, 2010, 2012) allows them in turn to suppress excitatory vagal output of the DMV (Herman et al., 2010). The resultant net result of suppressing this efferent vagal pathway would be a reduction in BW as evidenced previously by the weight-reducing effect of SDV in the VMH obesity syndrome, and at present by the *Mc4r*^−/−^ obesity phenotype model.

In trying to understand the mechanisms by which ablation of the vagus nerve results in such dramatic weight loss in *Mc4r*^−/−^ mice, we focused on the time course of weight loss in our animals taking cumulative FI into consideration in both the preventative and the reversal studies. During the first week after surgery, when animals were put back on a solid food diet, there was a reduction in FI in the vagotomized *Mc4r*^−/−^mice across both models (preventative and treatment) compared to controls (SS and PS groups). However, despite the fact that the reduction in FI did not persist throughout the post-surgical monitoring period (after week 1), it is important to note that SDV blunted any compensatory hyperphagia that may have occurred due to the state of negative energy balance (as characterized by decreases in both BW and FI in the first week) induced by SDV. Consistent with this, the PV group, in comparison to the PS group in the treatment arm of the study, did not over-eat relative to baseline levels in any of the experimental paradigms. The compensatory hyperphagia exhibited in the PS group during weeks 3 and 4 of the treatment arm of the study may be due to “dumping syndrome” or the accelerated emptying of gastric contents into the small bowel associated with the pyloroplasty drainage procedure (Bowers and Stockard, 1966). This compensatory hyperphagia is not present in the PV group; thereby, attesting to the effectiveness of SDV to exert a chronic anorexigenic effect.

Since FI was reduced in the first week and then normalized to that of controls in subsequent weeks, we decided to measure EE in the “treatment arm” of the study once a robust loss in weight (~17 g) had occurred in the PV group (that had undergone SDV). Both the non-normalized and normalized EE data indicate that the PV group did not exhibit the highest EE [kcal/h/kg] despite showing a robust weight loss. Rather, it was the PS group that displayed the highest EE, even though it showed no weight loss. This can be attributed to several factors. First, the PS group in the treatment phase had a higher FI relative to the PV group (Figure 3C). Second, animals with larger body mass are widely known to expend more energy as they have a higher basal metabolic rate (Felig et al., 1983; Arch et al., 2006).

The finding that the PV group did not exhibit the highest EE is not surprising in view of the role of *Mc4rs in EE (Ellacott and Cone, 2004; Cone, 2005)*. In diet-induced obese mice, signaling via *Mc4rs* in the cholinergic preganglionic neurons of the DMV has been reported to mediate the increase in EE seen after Roux-en-Y Gastric Bypass surgery (RYGB; Zechner et al., 2013). Furthermore, the *Mc4r* dependent increase in EE is thought to be the predominant mechanism responsible for post-surgical weight loss after RYGB (Zechner et al., 2013). While our findings are consistent with the lack of an increase in EE (in the PV group relative to the PS group) in *Mc4r*^−/−^ mice, SVD was still able to exert a robust weight-reducing effect; thus, underscoring the impact of the vagus nerve on the Mc4r obesity phenotype.

Despite the PV group not having the highest EE, our results show that RER in this group was significantly lower than that of the PS and SS treated controls during the dark and light cycles. RER, which normally displays a circadian rhythm, is associated with energy metabolism of carbohydrates and lipids (Dean et al., 2009; Speakman, 2013). A high RER value indicates a reduced use of fatty acid oxidation for daily EE, a condition associated with increased risk for weight gain and insulin resistance (Ravussin and Smith, 2002), whereas an RER approaching 0.7 indicates utilization of lipids as an energy source. The reduced RER in the PV group (between 0.80 and 0.85) in comparison to the SS and PS groups indicate that the PV group was shifting from a carbohydrate to that of fat-based metabolism at rest. Alternatively, preserved peripheral thermogenic mechanisms involving BAT-related fat oxidation or the thermogenic response to food at rest in these vagotomized animals (Andrews et al., 1985) may also explain the decrease in RER in the PV group. Moreover, these differential effects on fat and carbohydrate metabolism in the PV group maybe reflective of circulating insulin levels (Andrews et al., 1985). The decrease in RER in the PV group (relative to the SS and PS control groups) may indicate another potential mechanism by which vagotomy may lower BW in *Mc4r*^−/−^ mice. The LA in the PV group is significantly increased in the dark and light cycles when compared with the two control groups, indicating another potential mechanism of weight loss with increased physical activity. It is interesting however that LA is not correlated to EE across all groups with the PS group demonstrating increased EE but not a corresponding increase in LA.

While it appears that the reduction in RER and increase in LA could be contributing factors mediating SDV-mediated weight loss in the PV group, the corresponding anomaly in the EE data of the PS group to that of the PV group makes it difficult to draw any conclusions. It is quite conceivable that the loss of weight in the PV group may reflect some degree of carbohydrate malabsorption, which has been reported in patients undergoing vagotomy and pyloroplasty (Radziuk and Bondy, 1982). Nevertheless, whether changes in the various components of EE, changes in macronutrient absorption or even changes in intestinal transit in a non-specific manner are contributors to weight loss after SDV with pyloroplasty remains to be determined in future studies.

It should also be noted that the relation between variation in inter-individual levels of EE and weight gain/ or loss over long periods remains controversial. We measured 3 components of energy consumption (i.e., EE, RER, LA), which were collected over a short time-period (18–28 h) relative to the chronic nature of the study (involving cumulative changes in body weight over an extended period). Since these factors can vary substantially over time, measurement over a single sample period is not representative of the averages over the entire term of the study. This reduces the ability of our collective EE data to serve as an effective predictor of the mechanism of SDV-induced weight change that persists over a long time-period. Further indirect calorimetry studies of longer duration are required to determine the underlying mechanisms that account for the effect of SDV on weight loss.

In further trying to understand SDV-mediated weight loss, our data indicate that the weight loss associated with it has two components. An initial weight loss phase that is followed by a maintenance phase of the weight loss. This suggests that both autonomic and metabolic changes associated with SDV may alter the body set-point in two phases. The first phase may be associated with the initial weight loss due to reduced FI. It is conceivable that during early weight loss, SDV alters the set point due to reversal (or normalization) of the underlying hyperinsulinemia in *Mc4r*^−/−^ mice (Huszar et al., 1997; Ste Marie et al., 2000) as a result of denervation of the insulin-secreting cells of the pancreas, and presumably depletion of hepatic glycogen stores. The resultant increase in EE would facilitate decreases in adipose stores; thus, resulting in restored insulin sensitivity (Klaman et al., 2000). That this was not seen in our study may be due to EE data that represented the maintenance phase of the weight loss seen in the PV group. Therefore, it is likely that the PV group may have displayed the highest EE compared to controls if data were collected in the initial weight loss phase.

While the effect of SDV on BW in *Mc4r*^−/−^ mice is significant, it cannot be explained solely by differences in EE, RER, and LA as presented in this study. Since the vagus nerve is the major neuroanatomical link between the gastrointestinal (GI) tract and the brain, it is conceivable that potential peripheral mechanisms at the visceral target organs (in addition to the observed increase in EE) could account for the magnitude of effect on BW induced by SDV. In the GI tract, re-structuring of gut microbiota as a result of pH changes (Angelakis et al., 2013; Liou et al., 2013) due to surgical intervention have been shown to reduce BW, and therefore should be considered as a possible mechanism (Ionut and Bergman, 2011; Li et al., 2011).

In our study, intra-abdominal (mesenteric) and peri-renal fat deposits were significantly reduced in PV mice in comparison to SS and PS controls, which suggests a role for the vagus nerve in innervating, partitioning, or anabolic processing of WAT. This is particularly noteworthy in light of studies that show that parasympathetic innervation of subcutaneous and intra-abdominal WAT modulates insulin sensitivity, glucose, and free fatty acid metabolism (Kreier et al., 2002). Therefore, it is reasonable to assume that SDV reduces intra-abdominal WAT by removing the influence of anabolic processes, thus favoring metabolic pathways promoting weight loss. However, studies on vagal innervation of WAT (Kreier et al., 2002) have to be interpreted with caution as some studies have reported the absence of vagal projections to this tissue (Giordano et al., 2006).

In the “preventative” study, along with the anti-obesity effect of SDV, we observed a decrease in the snout-to-anus length, which was absent in animals in the “treatment study.” This is noteworthy as there are reports in the literature that show that mutations in the *Mc4r* gene are associated with accelerated linear growth, which is disproportionate to the degree of obesity present (Martinelli et al., 2011). In rats, vagotomy decreases the basal levels of both growth hormone (Al-Massadi et al., 2011) and insulin levels (see e.g., Lee and Miller, 1985) suggesting that in our study, SDV ‘blunted’ the increase in growth in part by attenuating both growth hormone and insulin levels. SDV also alters GI endocrine function that is required for maintaining energy homeostasis. For instance, vagotomy been shown to decrease and/or block the orexigenic effects of ghrelin (le Roux et al., 2005) presumably by denervation of the fundic cells responsible for the hormone's production.

Leptin-melanocortin signaling in the brain contributes to energy homeostasis in response to the adipostatic hormone leptin (Cone, 2005), but how this is affected by SDV needs to be considered. Sachot et al. (2007) reported that while vagal afferents do not constitute a considerable role in mediating the regulatory effect of leptin on FI, they acknoweldge that SDV surgery could mask the effects of leptin-mediated NTS activation via the efferent vagus nerve. This is likely as hypothalamic leptin signaling regulating insulin sensitivity is dependent on the hepatic branch of the vagus nerve (German et al., 2009). Therefore, it is conceivable that the weight loss associated with the SDV surgery ameliorates the underlying hyperleptinemia characteristic of the *Mc4r*^−/−^model (Itoh et al., 2011) via a vagal independent mechanism. Alternatively, leptin singaling that has been reported to contribute to the pathogenesis of the hepatic liver phenotype in *Mc4r*^−/−^mice (Itoh et al., 2011) is attenuated by SDV, thereby protecting against tissue damage. Further studies are needed to elucidate how dennervation of the vagus nerve leads to weight loss in *Mc4r*^−/−^ through changes in leptin signaling.

While SDV is an accepted method for evaluating the role of efferent vagus in controlling BW (Powley and Opsahl, 1974; Inoue and Bray, 1977; Cox and Powley, 1981b; Sclafani et al., 1981; Berthoud, 2008), it is nevertheless not an ideal one as it also eliminates afferent vagal fibers. These fibers are well-known to influence satiety and affect FI (Marsh et al., 1999b; Schwartz, 2000; Ritter, 2004; Peters et al., 2005; Berthoud, 2008; Campos et al., 2013, 2014), which may lead to an increase or maintenance of BW. However, in our study, BW was decreased as the result of SDV suggesting that effect was due to severance of vagal efferents and not afferents. A long-term effect of vagal efferents in the control of BW, as opposed to vagal afferents is consistent with those of vagal deafferentation studies (Chavez et al., 1997; Marsh et al., 1999a; Fox et al., 2001; Udit et al., 2017). For example, in a genetic mouse model of primary afferent neuron ablation, mice lacking large subsets of vagal afferent neurons such as those innervating the GI tract, displayed no differences in BW, FI, EE, or body composition compared to controls on chow diet (Udit et al., 2017). Additionally, while capsaicin-induced chemical ablation of visceral afferents results in overconsumption of unfamiliar palatable food, the effect is transient as it normalizes quickly to the control levels within 24 h (Chavez et al., 1997). Furthermore, afferent fibers show regeneration (albeit imperfectly) after 18 weeks subsequent to SDV, whereas the same is not evident for efferents (Phillips et al., 2003). In these studies, the SDV animals continue to lose weight (Phillips et al., 2003). Overall, our study and the aforementioned vagal deafferentation studies establish the importance of vagal efferents in long-term control of BW. These findings together with our data underscores the importance of parasympathetic preganglionic neurons in the brainstem in promoting anabolic aspects of energy homeostasis. We agree that the ideal design of our study would have been to use a pharmacological intervention to examine the effect of selective bilateral lesion of the dorsal motor nucleus of the vagus (DMV), the hindbrain nucleus containing the cell bodies of the preganglionic vagal neurons of the efferent pathway or by selectively sectioning efferent fibers as opposed to performing SDV. This would have selectively eliminated the efferent vagal fibers innervating peripheral organs such as the GI tract, liver, and pancreas, as well as offer pharmacological validation to the use of a surgical intervention. However, this is technically challenging due to the close proximity of DMV to the nearby nucleus of the solitary tract (NTS) and area postrema (Cruz et al., 2007), structures which also influence GI, liver, and pancreatic function (Woods and Porte, 1974; Sawchenko and Friedman, 1979; Gillis et al., 1989; Berthoud et al., 1991).

In summary, our data indicate that the hyperactive vagal efferent signaling underlying the hypothalamic obesity syndrome is also critical to the *Mc4r*^−/−^ obese phenotype, which is ameliorated by SDV.

## Author contributions

GD, RG, KLD, JV, and NS: conceived and designed the study; GD, JT, and NS: performed the experiments; GD, KRD, RG, and NS: analyzed data and prepared figures; GD, KRD, RG, and NS: wrote the manuscript with comments from all the authors. All authors approved the final version of the manuscript.

### Conflict of interest statement

The authors declare that the research was conducted in the absence of any commercial or financial relationships that could be construed as a potential conflict of interest.
